# Recent advances in the theoretical studies on the electrocatalytic CO_2_ reduction based on single and double atoms

**DOI:** 10.3389/fchem.2023.1172146

**Published:** 2023-03-28

**Authors:** Yuxiao Meng, Hongjie Huang, You Zhang, Yongyong Cao, Hanfeng Lu, Xi Li

**Affiliations:** ^1^ State Key Laboratory Breeding Base of Green−Chemical Synthesis Technology, College of Chemical Engineering, Institute of Industrial Catalysis, Zhejiang University of Technology, Hangzhou, China; ^2^ College of Biological Chemical Science and Engineering, Jiaxing University, Jiaxing, Zhejiang, China

**Keywords:** single-atom catalysts, double-atom catalysts, theoretical calculations, CO_2_RR, electrode potential, solvent effect

## Abstract

Excess of carbon dioxide (CO_2_) in the atmosphere poses a significant threat to the global climate. Therefore, the electrocatalytic carbon dioxide reduction reaction (CO_2_RR) is important to reduce the burden on the environment and provide possibilities for developing new energy sources. However, highly active and selective catalysts are needed to effectively catalyze product synthesis with high adhesion value. Single-atom catalysts (SACs) and double-atom catalysts (DACs) have attracted much attention in the field of electrocatalysis due to their high activity, strong selectivity, and high atomic utilization. This review summarized the research progress of electrocatalytic CO_2_RR related to different types of SACs and DACs. The emphasis was laid on the catalytic reaction mechanism of SACs and DACs using the theoretical calculation method. Furthermore, the influences of solvation and electrode potential were studied to simulate the real electrochemical environment to bridge the gap between experiments and computations. Finally, the current challenges and future development prospects were summarized and prospected for CO_2_RR to lay the foundation for the theoretical research of SACs and DACs in other aspects.

## 1 Introduction

Electrochemical technology shows significant promise for energy storage and addressing environmental issues. The massive consumption of fossil fuels has enormously increased carbon dioxide (CO_2_) in the atmosphere, accelerating the deterioration of the environment (Gao et al., 2016a; Cao et al., 2020a; Bai et al., 2022). Electrocatalytic carbon dioxide reduction reaction (CO_2_RR) directly converts CO_2_ into high-value chemicals, providing a favorable way to solve energy and environmental issues. However, the efficient activation of CO_2_ is a critical issue depending on the interaction between the catalyst surface and the CO_2_ molecules (Gong et al., 2019; Han et al., 2020a; He et al., 2020a). Therefore, various metal-based catalysts have been developed to accelerate the electrocatalytic CO_2_RR and achieve efficient CO_2_ conversion. Although bulk noble metal-based catalysts exhibit high catalytic activity, their large-scale production and practical applications are limited due to high cost and poor stability (Wu et al., 2016; Wang et al., 2019; Li et al., 2021a; Yang et al., 2022a). When the metal nanoparticles are reduced to nano clusters or even single atoms, the supported metal catalyst reaches an idealized state, which can greatly reduce the production cost of the catalyst and maximize the utilization rate of the precious metal (Ling et al., 2017; Lininger et al., 2021). Therefore, single-atom catalysts (SACs) have been used in a variety of reactions to promote the synthesis of high-value compounds and improve significant catalytic performance due to their unique electronic structure, high selectivity, homogeneous active sites and near 100% atomic utilization (Wang et al., 2020; Wan et al., 2022; Xu et al., 2022). However, the surface free energy of the SACs increases sharply with increased specific surface area, leading to easy agglomeration coupling and the formation of large clusters during their preparation and reaction. Therefore, double-atom catalysts (DACs) were proposed to make up for the deficiency of SACs. The increase of atomic load in the DACs results in multiple active central sites, and the synergistic effect, spacing enhancement effect and electron effect between diatomic sites help to regulate the electron distribution of the active sites, and effectively improve the catalytic performance (Song et al., 2020; Zhang et al., 2020; Hu et al., 2022). Therefore, SACs and DACs are increasingly popular in oxygen reduction reaction (ORR) (Cao et al., 2020b; Talib et al., 2021; Zhao and Liu, 2021; Chen et al., 2022), oxygen evolution reaction (OER) (Zhou et al., 2020a; Zeng et al., 2020; Xue et al., 2021), nitrogen reduction reaction (NRR) (Lv et al., 2021; Xu et al., 2021; Niu et al., 2022), hydrogen evolution reaction (HER) (Gao et al., 2016b; Zhang et al., 2017), and CO_2_RR due to their unique properties (Cao et al., 2022a; Chou et al., 2022; Sun et al., 2023).

Density functional theory (DFT) is a powerful tool to understand materials and catalytic reaction elementary steps and mechanisms at the atomic scale. Additionally, DFT can explore the electronic structure of the catalyst and directly identify the active sites compared with the experimental method (Li et al., 2020a; Lininger et al., 2021; Yang et al., 2022b). Furthermore, it reveals the intrinsic nature of the activated CO_2_ molecule to understand the CO_2_RR mechanism. The influence of solvation and ionic effects on CO_2_RR at the solid-liquid interface is relatively complex and relies on a first-principles approach to provide theoretical insights into its mechanism. Therefore, the theoretical computational methods provide fundamental guidance and predictions to rationalize catalyst models.

Various studies and reviews on the synthesis, characterization, and electrocatalytic applications of SACs and DACs have been published. This review discussed the unique internal performance of SACs and DACs and reviewed their catalytic mechanism on CO_2_RR from the perspective of theoretical calculations. This review explored the influence of solvent and surface charge on electrocatalytic CO_2_RR. Finally, the challenges and perspectives for future studies on CO_2_RR were also provided.

## 2 SACs

In 2011, Zhang et al. synthesized the Pt_1_/FeOx catalyst and first proposed the concept of SAC (Qiao et al., 2011). In the case of the supported catalysts, the catalytic activity of metal catalysts is closely related to their particle size. The specific surface area increases dramatically when the dispersion of nanoparticles reaches the single atom size, maximizing the utilization of metals and significantly reducing the cost of precious metal catalysts (Hossain et al., 2020; Pu et al., 2020). Multiple active sites are exposed on the catalyst surface with uniform contact between the active site and the support, significantly improving the activity and selectivity of the catalytic reaction (Lu et al., 2019). SACs often exhibit different activity and selectivity compared to conventional nano-catalysts due to the coordination number of central atoms and the electronegativity of neighboring atoms (Cepitis et al., 2021; Huang et al., 2023; Li et al., 2023). The strong interaction between the isolated atoms and the carrier can induce charge redistribution on the carrier surface, improving the reactional intrinsic activity and stability of the catalyst. SACs can modify the adsorption and desorption selectivity of the active components of the catalyst in different molecules, affecting the reaction kinetics.

### 2.1 Strong covalent metal–support interaction

**FIGURE 1:**
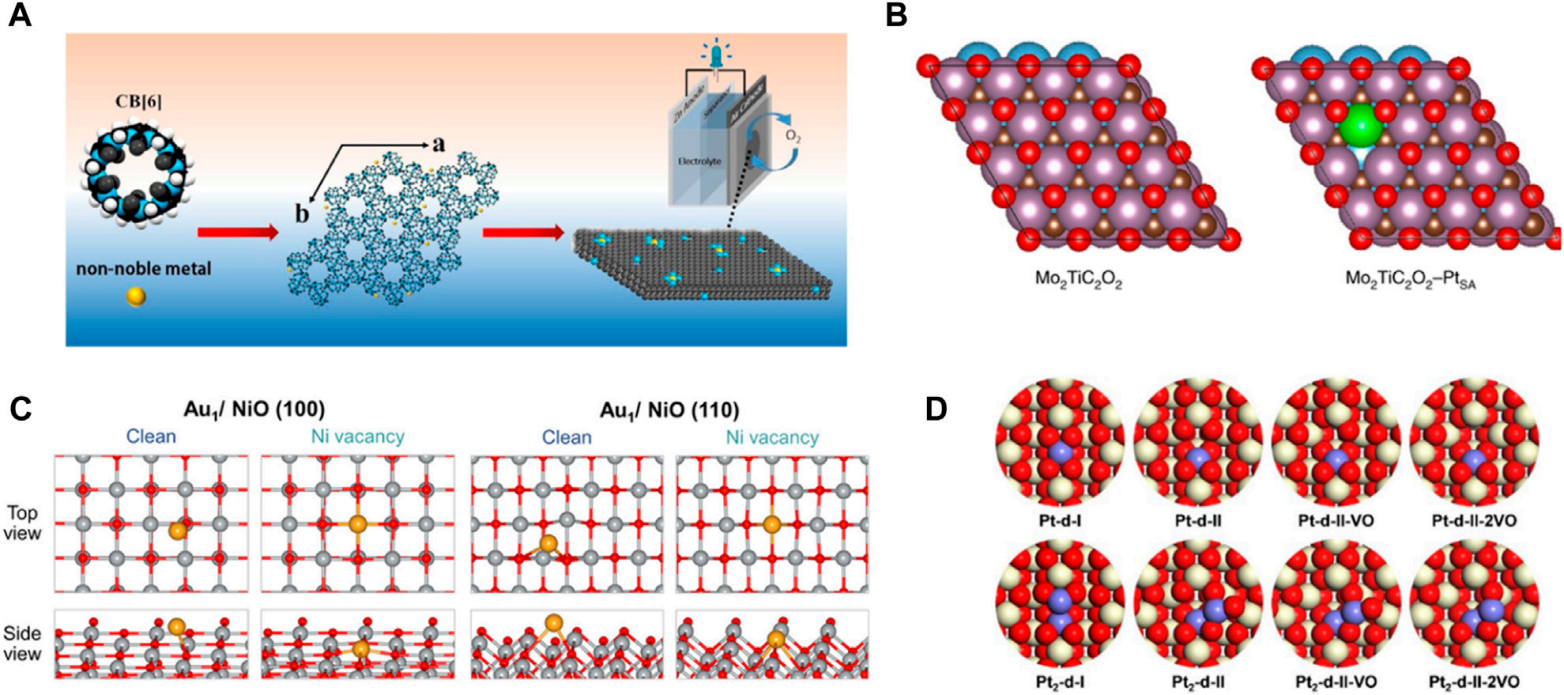
| The interaction of metals with different supported materials. (A) Schematic diagram of non-noble metal atom mixed with CB (He et al., 2020a) for ORR electrocatalysis. (Figure used with permission from Cao et al., 2021) **(B)** Top view of Mo_2_TiC_2_O_2_ and Mo_2_TiC_2_O_2_-Pt_SA_ catalyst configurations. Color codes: Ti, blue; Mo, purple; Pt, green; C, brown; O, red. (Figure used with permission from Wang et al., 2018) **(C)** Geometry of Au single atoms deposited on clean and defective NiO(100) or NiO(110) planes. Gray, Red and Yellow represent Ni, O, Au atoms, respectively. (Figure used with permission from Murayama et al., 2022) **(D)** Local structure of Pt atoms doped into CeO_2_ (110). White, blue, Red and lattice represent Ce, O, Pt atoms, respectively. (Figure used with permission from Su et al., 2021).

Numerous efforts have been made to increase the surface area by reducing the size of metallic nanomaterials to atomic levels, adequately exposing many active sites, and improving the electrocatalytic performance (Konsolakis and Ioakeimidis, 2014; He et al., 2023). However, due to the extremely high surface energy of single metallic atoms, they are highly susceptible to migration and aggregation under synthetic and catalytic conditions. Therefore, the migration of single atoms is often confined using the strong interactions between the isolated metal atom and the support. Different properties of the support materials affect the coordination number, steric environment, and chemical bonding, resulting in SACs with different electronic and morphological structures (Yang et al., 2020). Most single metal atoms can be immobilized on the carbon material by coordination with nitrogen atoms (M-Nx), which maximizes the atomic utilization of the metal atoms (Figure 1). Moreover, the low coordination environment allows firm anchorage of metal atoms on the carrier with high stability and electrocatalytic activity (Tang et al., 2022). Cao et al. designed a series of M-N-C SACs with macrocycle cucurbit (He et al., 2020a) uril (CB (He et al., 2020a)) self-assembly as carbon precursors. The Fe-loaded N-doped holey carbon single-atom electrocatalyst (Fe-NHC) exhibited higher stability compared to SACs of cobalt (Co) or nickel (Ni) and showed higher activity for ORRs under alkaline conditions (Figure 1A) (Zhang et al., 2021a). Feng et al. synthesized carbon nanosheets embedded with isolated copper atoms coordinated with N(Cu-N-C-800). The catalysts exhibited excellent stability after 20 consecutive cycles and facilitated the conversion of nitrate to ammonia (NH_3_) and nitrogen (Zhu et al., 2020). MXene materials (two-dimensional) with a graphene-like structure can control their structure and properties by changing the ratio of M and X elements, regulating their electrical conductivity and carrier mobility, and the MXene materials have high mechanical stability. They improve the activity of SACs as catalyst carriers and exhibit strong catalytic reduction abilities (Zhang et al., 2021b). Wang et al. showed that single platinum (Pt) atoms occupied the Mo vacancies in MXene, and it were connected with the carbon (C) atoms to form three Pt-C bonds (Zhang et al., 2018). The strong covalent interactions between the positively charged Pt atoms and MXene improved the stability of the catalyst and the catalytic ability for HER with low overpotentials of 30 and 77 mV to achieve 10 and 100 mA cm^−2^ (Figure 1B). SACs based on oxide supports are also used in various reactions (Zhou et al., 2020c; Han et al., 2021). The defect sites and -OH groups on the surface of oxides provide the possibility of anchoring single atoms. The electron transfer makes the strong interaction between oxides and single atoms, which improves the mechanical and thermal stability of SACs. It was conducive to the application in electrocatalytic reactions (Han et al., 2020b; Lang et al., 2020) In Y_2-x_Co_x_Ru_2_O_7−δ_, the introduction of Co atoms leads to a redistribution of charge between Ru and Co atoms, resulting in ultra-high OER activity. In addition, Co substitution also creates oxygen vacancy, which leads to the rapid transfer of OER charge. The strengthening of the bond hybridization between the d orbitals of Y and Ru and the 2p orbitals of O further enhances the chemical stability of the catalyst (Qin and Su, 2021). Murayama et al. reported a nickel oxide-supported single gold atom catalyst (Au_1_/NiO) for carbon monoxide (CO) oxidation reaction (Mochizuki et al., 2022). The adsorption energies of gold (Au) atoms dispersed on nickel oxide (NiO) surfaces and Au atoms dispersed on NiO surfaces with Ni vacancies were calculated using DFT. Subsequently, it was observed that the formation of single Au atoms was more favorable on the NiO surfaces with Ni vacancies than on clean NiO surfaces, single Au atoms were cationic on the NiO surfaces. The results were consistent with the experimental observations. Besides, Au_1_/NiO showed high catalytic stability to the CO oxidation reaction (Figure 1C). Zhang et al. used Mo atoms to regulate the oxidation state of transition metals in perovskite oxides by substituting Ni sites as highly selective and active catalysts for efficient 2e^−^ ORR production of H_2_O_2_ (Han et al., 2023). Cerium oxide (CeO_2_) can stabilize single metal atoms due to its high vacancy density. Su et al. used DFT to calculate all the configurations of the single-atom Pt/CeO_2_ (110) catalyst (Qin and Su, 2021). They found high stability for structural models where cerium cations were doped with one or two Pt atoms, which was ascribed to the formation of square-planar oxygen coordination (Figure 1D). Therefore, rationally selecting the support and designing catalysts with stably anchored isolated atoms are crucial for electrocatalytic reactions in the future.

### 2.2 Activity of SACs

**FIGURE 2:**
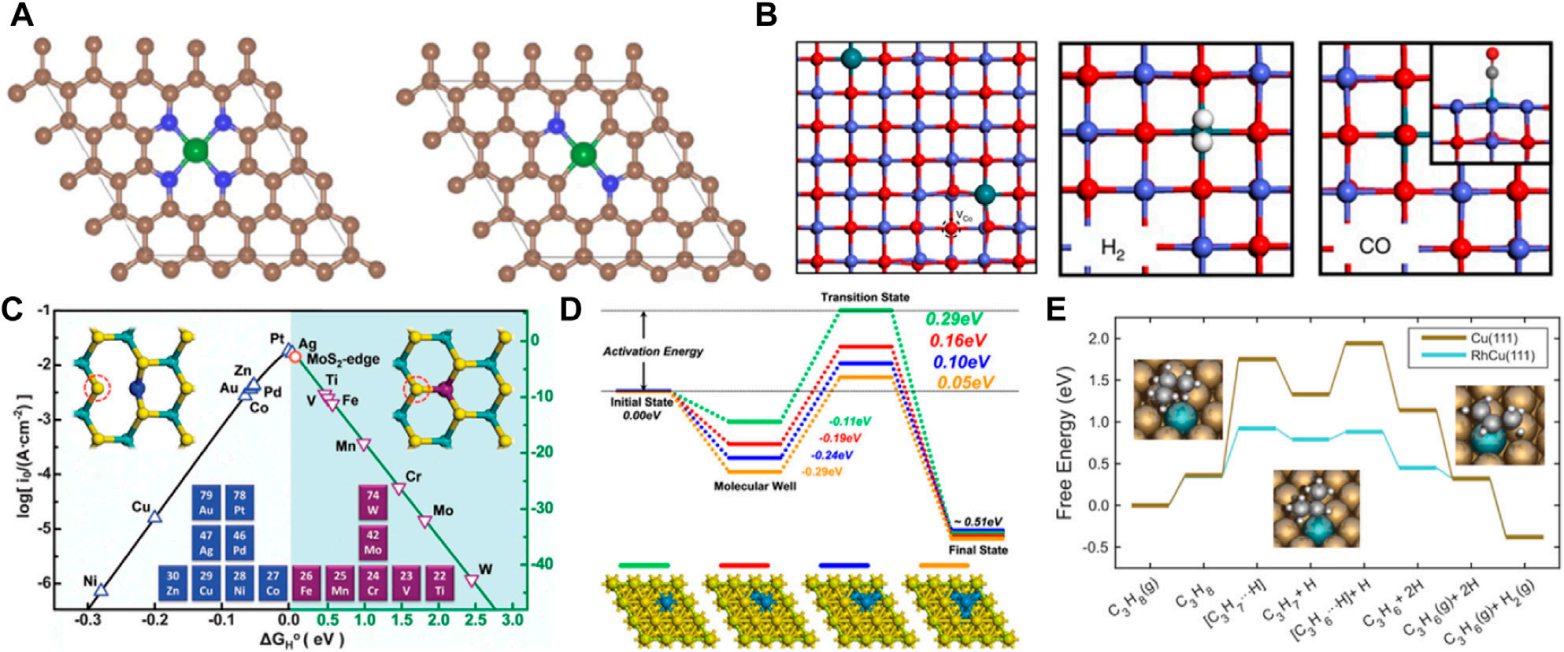
**(A)** Ni–SAC model diagram containing Ni–N_4_ and Ni–N_2_C_2_ moieties as active sites for CO_2_RR (figure used with permission from Luo et al., 2020). **(B)** The model of Rh_1_/CoO and the adsorption configurations of H_2_ and CO on Rh_1_/CoO (figure used with permission from Zeng et al., 2016) **(C)** The volcano curve relation between currents [log(*i*_0_)] and △G_H_^。^. The inserted diagram points to different doped MoS_2_ configurations (figure used with permission from Luo et al., 2013). **(D)** Energy profiles of H_2_ dissociation and structure diagram of Pd-doped Cu(111) surfaces (figure used with permission from Luo et al., 2013). **(E)** Free-energy diagram of RhCu (111) SAA and Cu(111) for the formation of propylene. (Figure used with permission from Sykes et al., 2021).

Improving catalytic activity has been a key problem that has been studied by many researchers. Although the intrinsic activity of the catalyst can be affected by the interaction between the carrier and single atoms, the abundance of active sites on the catalyst surface is an important factor for effectively activating the reactants (Hou et al., 2023; Liu et al., 2023). Isolated metal atoms in SACs are often considered the main active sites (Figure 2). However, increasing the density of metal atoms and the number of active sites to achieve the target chemisorption of the reactants is an important indicator for improving the catalytic reaction activity (Zhang et al., 2021c). Theoretical studies have indicated that the activity of Ni−N−C for CO_2_RR changes as the coordination elements (N, C) vary, such as Ni−N_4_, Ni−N_3_C_1_, Ni−N_2_C_2_, Ni−N_1_C_3_, and Ni−C_4_ (Hossain et al., 2020). The CO stretch for CO bound to Ni was 1985 cm^−1^ at −1.0 V on the Ni–N_2_C_2_ site, which agrees best with the experimental results. It showed the best catalytic activity and selectivity. Furthermore, single metal atoms embedded into the carrier affect the surrounding carrier atomic sites and can act as active sites to play a synergistic catalytic role and improve the catalytic activity (Figure 2A). Zeng et al. found that isolated rhodium (Rh) atoms aggregated clusters of different atomicity by increasing Rh mass loading. Rh/CoO promoted the adsorption and activation of propylene in the hydroformylation of propylene (Wang et al., 2016). Due to the structural reconstruction of Rh single atoms in Rh/CoO, propylene and CO were trapped by Rh_1_, and the neighboring oxygen atoms of CoO provided adsorption sites for hydrogen (H) atoms, synergistically catalyzing the promotion of the hydroformylation of propylene (Figure 2B). Theoretical computations revealed that an increased content of single Pt atoms loaded on molybdenum disulfide (MoS_2_) affects the activity of the adjacent surface S atoms, increasing the activity of the catalytic reaction (Figure 2C) (Deng et al., 2015). In single-atom alloy (SAA) catalysts, composed of bimetallic and polymetallic complexes, one type of metal atom is atomically dispersed on the other metal material (Zhang et al., 2021d). The active sites are generated by the strong metal interaction (alloying bonding) between the isolated-single atom and the metal carrier material. Luo et al. found that ensembles composed of the Cu (111) surface and the palladium (Pd) atoms subsurface were considered active sites to effectively reduce the energy barrier of H_2_ dissociation through theoretical calculations (Fu and Luo, 2013). Subsequently, this can be employed to adjust the catalytic activity of SACs by doping other atoms in the subsurface layer (Figure 2D). In a newly reported study, it was suggested that the SAA catalyst with Rh atoms dispersed in Cu had a very low kinetic barrier in the process of dehydrogenation and formation of propylene (Hannagan et al., 2021). Additionally, it showed strong activation ability for the C-H bond. Therefore, the activity of SACs directly affects the reaction route and different activation mechanisms (Figure 2E). It guides the further designing of SACs by increasing the density of single-atom metals and the number of active sites.

### 2.3 Selectivity of SACs

**FIGURE 3:**
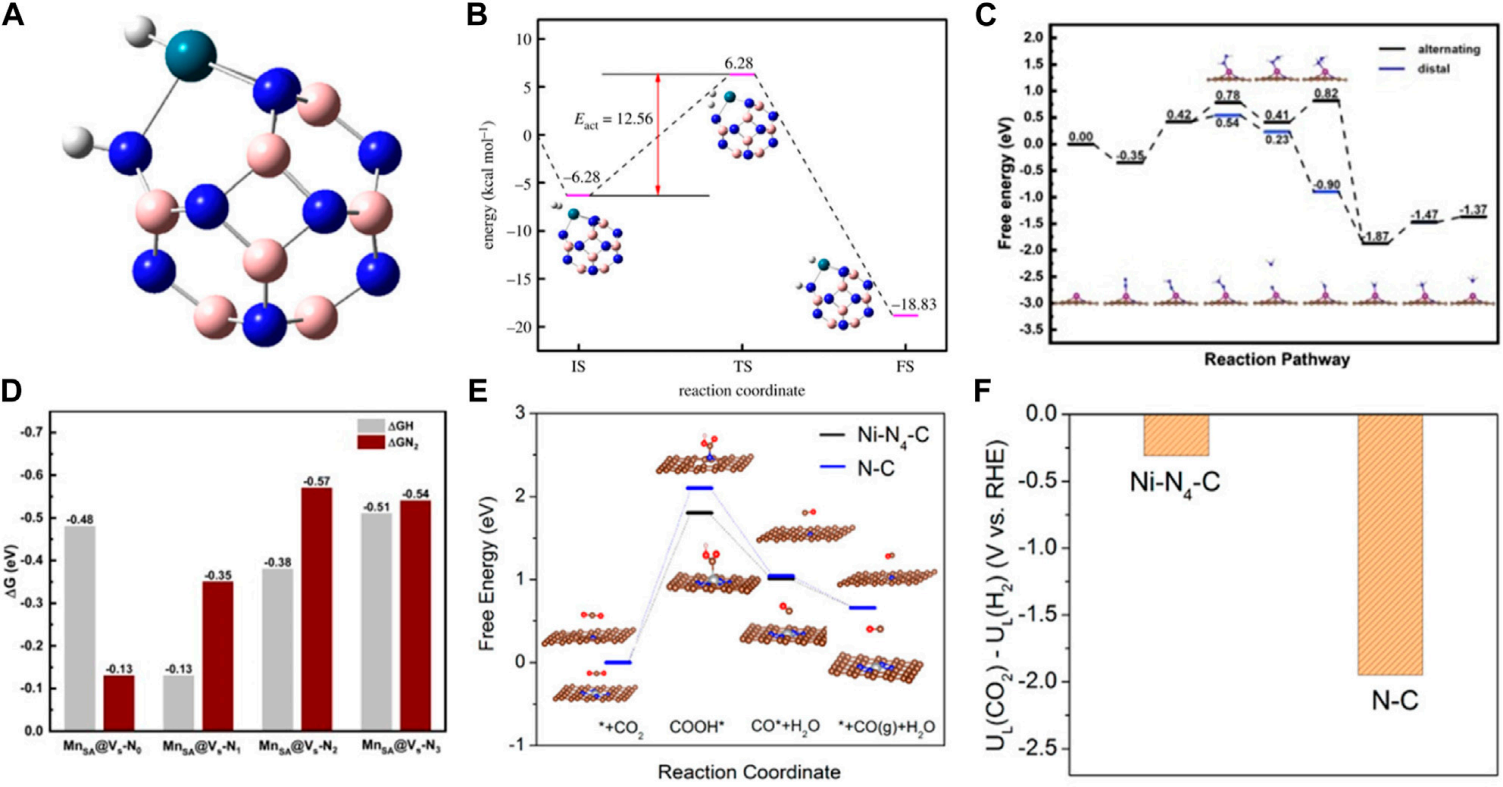
**(A)** The stable structures of H_2_ absorbed on B_11_N_12_Pd SAC. **(B)** Energy barrier diagram of the B_11_N_12_Pd SAC for hydrogenation reaction and the schematic diagrams of initial (IS), transition (TS) and final (FS) states. White spheres: H atoms, pink spheres: B atoms, blue spheres: N atoms, blue-green spheres: Pd. (Figure used with permission from Kang et al., 2017) **(C)** Free-energy diagrams for the NRR on Fe-N/C. **(D)** The difference between the free energy of H and N_2_ atom (figure used with permission from Zhao et al., 2020) **(E)** (a) The free energy diagram for CO_2_RR. **(F)** Limiting potential difference for CO_2_RR and HER on Ni-N_4_ catalyst (figure used with permission from Xie et al., 2017).

The reaction process is often accompanied by the occurrence of side reactions. Therefore, product selectivity is an important parameter in evaluating catalyst performance. Product selectivity is affected by the characteristics and conditions of the reactions. In addition, it is related to the catalyst (Li et al., 2017a; Ying et al., 2021; Zhang et al., 2022a). SACs exhibit high catalyst selectivity due to the unsaturated coordination of the active centers. The theoretical calculations showed that the charges were redistributed by replacing a B site in the B_12_N_12_ nanocage with a Pd atom (Gong and Kang, 2018). Moreover, the electrons were found to accumulate around the Pd atom in B_11_N_12_Pd. H_2_ was adsorbed and dissociated on single Pd atoms to form the B_11_N_12_Pd(2H) dihydride complex and then hydrogenated with C_2_H_2_ on the B_11_N_12_Pd SACs. The energy barrier was as low as 26.55 kcal mol^−1^, and it had higher selectivity than many bimetallic alloy monatomic catalysts (Figures 3A, B). Zhao et al. constructed 11 kinds of SACs by modulating the coordination environment of Mn atom and graphene substrate for NRR (Gao et al., 2020). MnSA@V_s_-N_1_ promoted the conversion of N_2_ to NH_3_ at 0.77 eV by distal mechanism, inhibiting the occurrence of HER (Figures 3C, D). The lower reaction free energy in the HCOO* formation (ΔG_HCOO*_) made it the main product on the atomically dispersed In^δ+^−N_4_ (Shang et al., 2020) and Sb-N_4_ SACs (Jiang et al., 2020). However, the HER pathway had much higher free energy than HCOO* formation, exhibiting excellent selectivity. The catalyst selectivity is evaluated with appropriate descriptors. Considering that H_2_ evolution was the most competitive in CO_2_RR, the difference between thermodynamic limiting potentials for CO_2_RR and H_2_ evolution was a proposed descriptor to evaluate the high selectivity of CO_2_RR to CO on Ni-N_4_ catalyst (Figures 3E, F) (Li et al., 2017b). Therefore, constructing correlations between selectivity and electronegativity, overpotential, and charge number are very useful for evaluating catalytic performance. Additionally, the theoretical designs provide a great basis for developing the experiments.

## 3 Applications of SACs for CO_2_RR

### 3.1 Noble metal SACs

**FIGURE 4:**
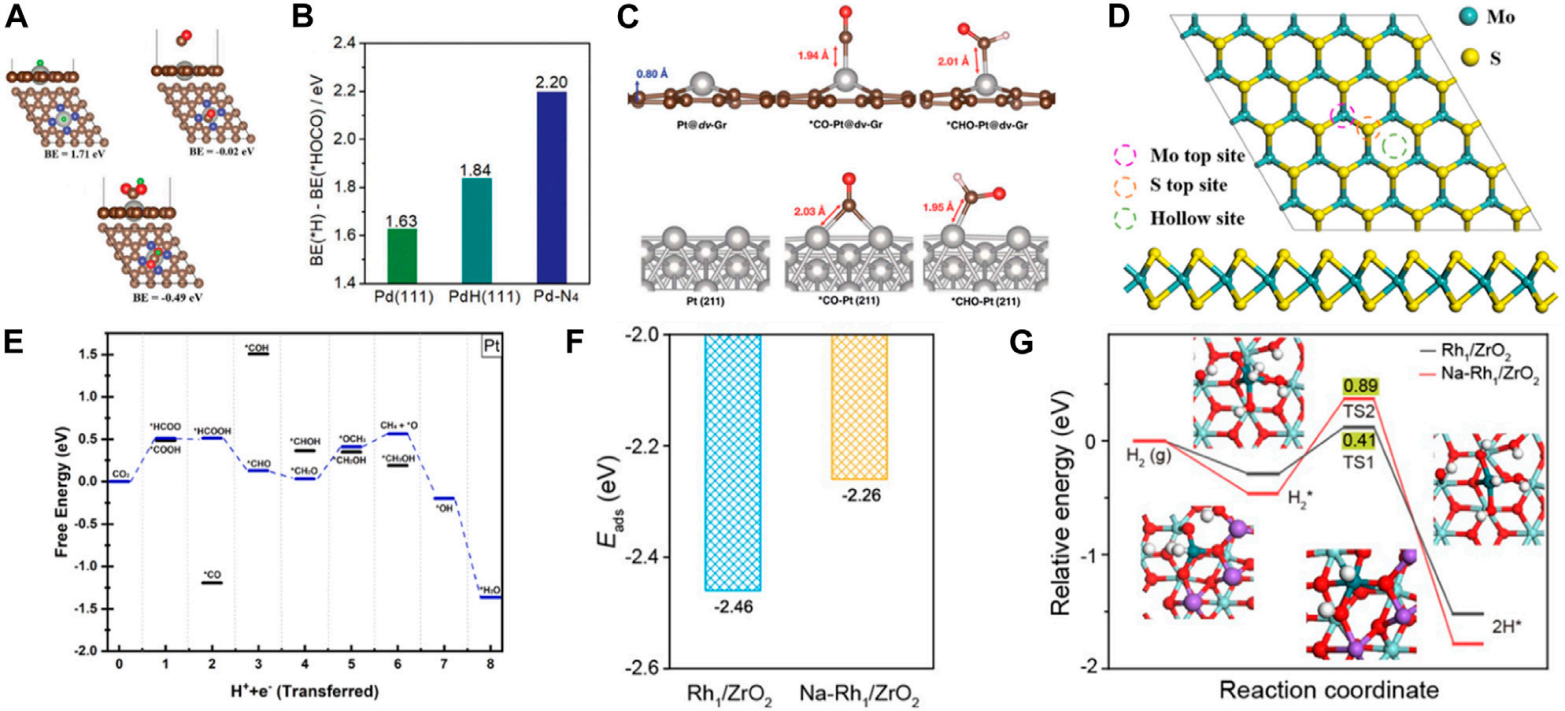
**(A)** The optimized structures and calculated binding energies of *H, *CO, and *HOCO adsorbed on a Pd-N_4_ catalytic by DFT calculations. Gray, Pd; brown, C; blue, N; red, O; green, H. **(B)** The binding energies difference of *H and *HOCO on Pd(111), PdH(111), and Pd-N_4_ (figure used with permission from Chen et al., 2020a). **(C)** The optimized configurations of *CO and *CHO adsorbed on Pt@dv-Gr and Pt(211) (figure used with permission from Jung et al., 2017). **(D)** The top views and side views of the MoS_2_ nanolayers. **(E)** Free energy diagrams for the CO_2_RR on Pt@MoS_2_. (Figure used with permission from Zhao et al., 2022a). **(F)** The adsorption energies of CO on Rh_1_/ZrO_2_ and Na-Rh_1_/ZrO_2_. (Figure used with permission from Lu et al., 2022). **(G)** The energy profiles of H_2_ reaction and the structures of the optimized intermediates on Rh_1_/ZrO_2_ and Na-Rh_1_/ZrO_2_. (Figure used with permission from Lu et al., 2022).

Noble metal SACs offer the benefits of high conductivity, a large active surface area, vacancies in the d orbitals, and the ability to adsorb/stabilize reactants and intermediates. Therefore, noble metal SACs are extensively utilized in electrocatalytic CO_2_RR. Chen et al. incorporated atomic Pd into N-doped carbon support to improve the CO_2_RR activity (He et al., 2020b). The binding energy of the intermediates, *H and *HOCO, on Pt-N_4_ were weaker than on Pd/PdH. However, the formation of *HOCO was more favorable than *H through the difference computed in their binding energies. Furthermore, in a realistic electrocatalytic environment, the intermediate *HOCO can enhance its stability on Pd-N_4_ by forming H bonds with water molecules. Therefore, Pt-N_4_ was the most likely to obtain CO without the formation of palladium hydride (PdH). The atomically dispersed Pd facilitated the desorption of weakly bound *CO and enhanced electrocatalytic CO_2_ reduction capability (Figures 4A, B). Since a single Pt atom can remarkably improve the CO_2_RR activity of graphene, it was anchored on defective graphene with double vacancies to form an atomically coordinated Pt@dv-Gr catalyst, achieving high selectivity and activity of CO_2_RR to form CH_3_OH (Back et al., 2017). Moreover, the weak binding energy of *CO on Pt@dv-Gr promotes protonation to form *CHO species. Pt fills the bonding orbital *via* orbital mixing due to single occupancy in the *d*_xz_ orbital that can interact with the C(*p*_z_) orbital of *CHO during the *CHO binding, leading to CO_2_ reduction to produce CH_3_OH at the low limit potential (*U*_L_) of −0.27 eV on Pt@dv-Gr (Figure 4C). Zhao et al. reported single Pt atoms supported on MoS_2_ nanolayers as CO_2_RR catalyst by DFT calculations (Ren et al., 2022). Pt@MoS_2_ were selective catalysts for CO_2_RR to produce CH_4_ with a *U*_L_ of −0.50 eV (Figures 4D, E). Poater et al. reported that single atom Ag was anchored on g-C_3_N_4_ support (Ag_1_@ g-C_3_N_4_) as a SACs, showing different activity for Cu_1_@ g-C_3_N_4_ (Posada-Pérez et al., 2023). The CO formation process requires to overcome the thermodynamic barrier with a limiting potential of −0.55 V. The desorption energy of CO was 0.56 eV, lower than that of Cu_1_@g-C_3_N_4_ catalyst. The most favored reaction pathway was the formation of HCOOH product with a low potential of −0.37 V. In addition, H_ads_ on the catalyst Ag_1_@ g-C_3_N_4_ are very unstable compared to CO_2_ adsorption. Therefore, Ag_1_@ g-C_3_N_4_ catalyst promotes the generation of HCOOH. Liu et al. study the structure of Ru/In_2_O_3_ catalyst and the intermediates and transition states of CO_2_ hydrogenation to methanol by DFT. The results not only confirm that the strong metal-support interaction improves the catalyst stability, but also show obvious charge transfer and rearrangement between Ru and In_2_O_3_ (Wu et al., 2021). The CO_2_ molecule was first activated at the oxygen site of the metal support interface, and then directly dissociated into CO*, the CO_2_ molecule was gradually hydrogenated until methanol was formed, and finally desorbed from the catalyst surface. The Ru/In_2_O_3_ catalyst showed high catalytic activity and stability. Lu et al. found that electron promoter Na^+^ enhances the electron interaction between the neighboring Rh_1_ and ZrO_2_ carriers and promotes electron transfer from Rh to ZrO_2_ carriers (Li et al., 2022a). It not only weakens the adsorption of CO on Rh_1_/ZrO_2_, but also reduces the activation ability of H_2_. This can promote the desorption of the reaction product CO, avoid the deep hydrogenation of CO products to CH_4_, and achieve high CO selectivity (Figures 4F, G).

### 3.2 Non-noble metal SACs

**FIGURE 5:**
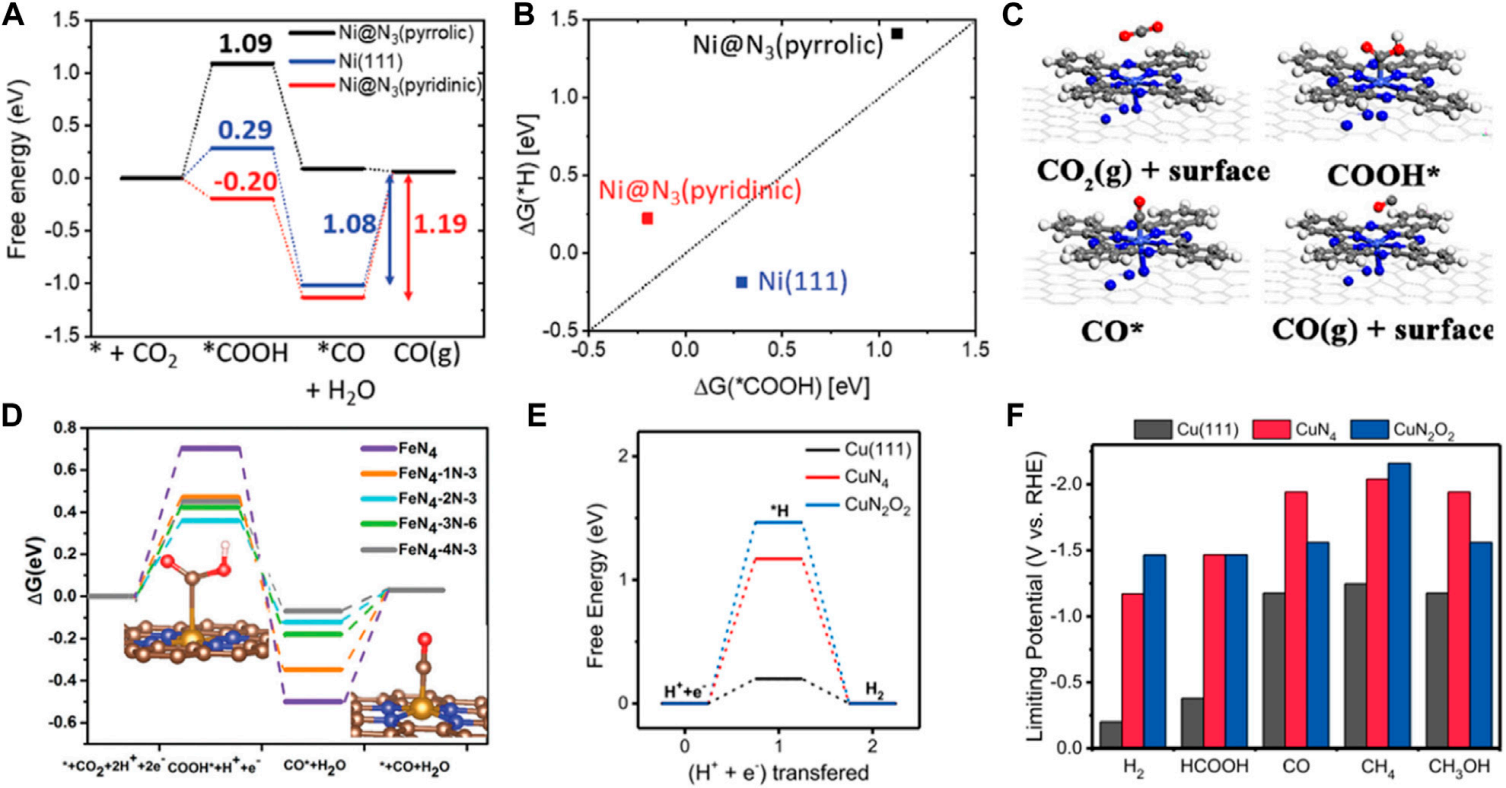
**(A)** Free energy diagram of electrochemical CO_2_ reduction to CO on Ni@N_3_ (pyridinic), Ni(111) and Ni@N_3_(pyrrolic). **(B)** The linear relationship between ΔG(*H) and ΔG(*COOH) (figure used with permission from Sun et al., 2019) **(C)** Optimized structures of intermediates for CO_2_RR (figure used with permission from Li et al., 2018) **(D)** Free energy diagram and intermediates geometries for electrochemical CO_2_ reduction to CO on Fe–N_4_ moieties embedded on carbon sheets. Gray, C; Orange, Fe; Blue, N; Red, O; light white, H. (Figure used with permission from Tang et al., 2022) **(E)** Free energy diagram of hydrogen evolution on the CuN_2_O_2_(Cu-CDs), CuN_4_, and Cu(111). **(F)** The limiting potentials of the all products on the CuN_2_O_2_, CuN_4_, and Cu(111) (figure used with permission from Zhu et al., 2021).

Non-noble metal catalysts with the transition metal coordination on carrier *via* defect engineering have also been reported for CO_2_RR with excellent catalytic performance. In a previously reported study, seven metals were atomically dispersed in a tungsten ditelluride monolayer (M@WTe_2_) for CO_2_RR using DFT calculations (Zhang et al., 2022b). Subsequently, Ni@WTe_2_ had the highest adsorption capacity for *CO and *HCOOH near the Fermi level and a suppressed competing reaction which led to higher activity and selectivity. The Gibbs free energy of CO_2_RR also demonstrated that the thermodynamic energy barrier of Ni@WTe_2_ was only 0.11 eV. Therefore, Ni@WTe_2_ was a promising electrocatalytic material for CO_2_RR. Sun et al. constructed the Ni–N_3_ (pyridinic) and Ni–N_3_ (pyrrolic) structures incorporated in different N-doped carbon supports to achieve high selectivity and activity of CO_2_RR to produce CO (Fan et al., 2019). DFT calculations showed that Ni–N_3_ (pyridinic) had low free energy for *COOH and *CO formation. The active site of Ni@N_3_ (pyridinic) was easily poisoned by *CO due to strong *CO adsorption. On the contrary, Ni-N_3_ (pyrrolic) was more likely to obtain CO. The free energy value for *COOH formation on Ni@N_3_ (pyrrolic) was lower than that reported for Ni-N_4_. Therefore, the catalytic activity for the Ni@N_3_ (pyrrolic) site may be better than the Ni@N_4_ site (Figures 5A, B). Li et al. designed atomically dispersed Co-N_5_ sites anchored on polymer-derived hollow N-doped porous carbon spheres (HNPCSs) for efficient CO_2_ reduction to produce CO (Pan et al., 2018). The Co-N_5_ site was the active center for CO_2_ reduction to produce CO. The thermodynamic barrier for the key COOH* formation was −0.28 eV on Co-N_5_/HNPCSs, and CO desorption ability was increased than that of the CoPc catalyst, exhibiting a higher CO_2_RR activity. Moreover, single Fe atoms can also significantly increase the CO_2_RR activity (Figure 5C). Tang et al. reported an atomically dispersed Fe-coordinated N-doped carbon catalyst that enhanced CO_2_ reduction to produce CO with high activity (Takele Menisa et al., 2022). The neighboring graphitic N transferred more electrons to the intermediate COOH*, increasing COOH* adsorption strength. However, the neighboring graphitic N transferred a reduced number of electrons between CO and the catalyst, promoting CO desorption. Therefore, the origin of the high activity was attributed to the synergistic effects between FeN_4_ and the neighboring graphitic N. The Fe-N_5_ sites were more conducive to producing CO than FeN_4_ (Figure 5D). Wu et al. reported the incorporation of Fe–N_5_ sites in the defect-rich porous carbon nanofiber (Fe-N_5_/DPCF) catalyst for CO_2_RR (Li et al., 2022b). The coupling of Fe-N_5_ with defect carbon (DC) substrate provided active sites for CO_2_ adsorption and activation. DC, Fe-N_5_/C, Fe-N_4_/C, and Fe-N_5_/DC were also studied to explore the activity and selectivity of CO_2_RR. The energy barrier of CO desorption on Fe-N_5_/DC was significantly lower than that of Fe-N_4_/C, suggesting high CO_2_RR activity. Moreover, the positive limiting potential differences between CO_2_RR and HER indicated the highest selectivity of Fe-N_5_/DPCF. Furthermore, Cu-based catalysts are unique catalysts capable of reducing CO_2_ to a range of hydrocarbon compounds with excellent selectivity. Zhu et al. performed DFT calculations and found that the N and O-coordinated single Cu atom catalyst (CuN_2_O_2_) had high selectivity and activity for CO_2_ reduction to produce CH_4_ (Cai et al., 2021). The positive charge of Cu in CuN_2_O_2_ was lower than in CuN_4_, resulting in a low-valence oxidation state of Cu. The energy barriers of intermediates *COOH and *COH in CuN_2_O_2_ were reduced, and HER was well-inhibited (Figures 5E, F). Zheng et al. demonstrated that the binding energies of CO in the two adjacent Cu–N_2_ sites were stronger than at the Cu-N_4_ sites, which was conducive to the formation of C_4_H_4_ through C-C coupling (Guan et al., 2020a). However, the isolated single-atomic Cu sites also had high activity and selectivity for CH_4_. It was found that the single tungsten (W) atom could improve the CO_2_RR activity of In_2_O_3_ (Zou et al., 2022). The single W atom was embedded on the In_2_O_3_ (111) substrate containing oxygen vacancies to form an atomically coordinated W-In_2_O_3__D model. The oxygen vacancy could provide more electrons for activating CO_2_. Additionally, W induced a higher degree of electron distribution in oxygen vacancies, resulting in the easier formation of the HCOO* intermediate and breaking of the C-O bond in the H_2_COOH* intermediate. Therefore, the generation of methanol products was significantly easier on the W-In_2_O_3__D catalyst. Xu et al. introduced single vanadium (V) atom into bismuth oxide (Bi_2_O_3_) to enhance CO_2_ conversion to formic acid (Zhang et al., 2022c). The redistribution of electrons on the Bi_2_O_3_ surface promoted CO_2_ adsorption and activity due to the introduction of the V atom, improving the selectivity of formic acid.

### 3.3 Metal-free SACs

**FIGURE 6:**
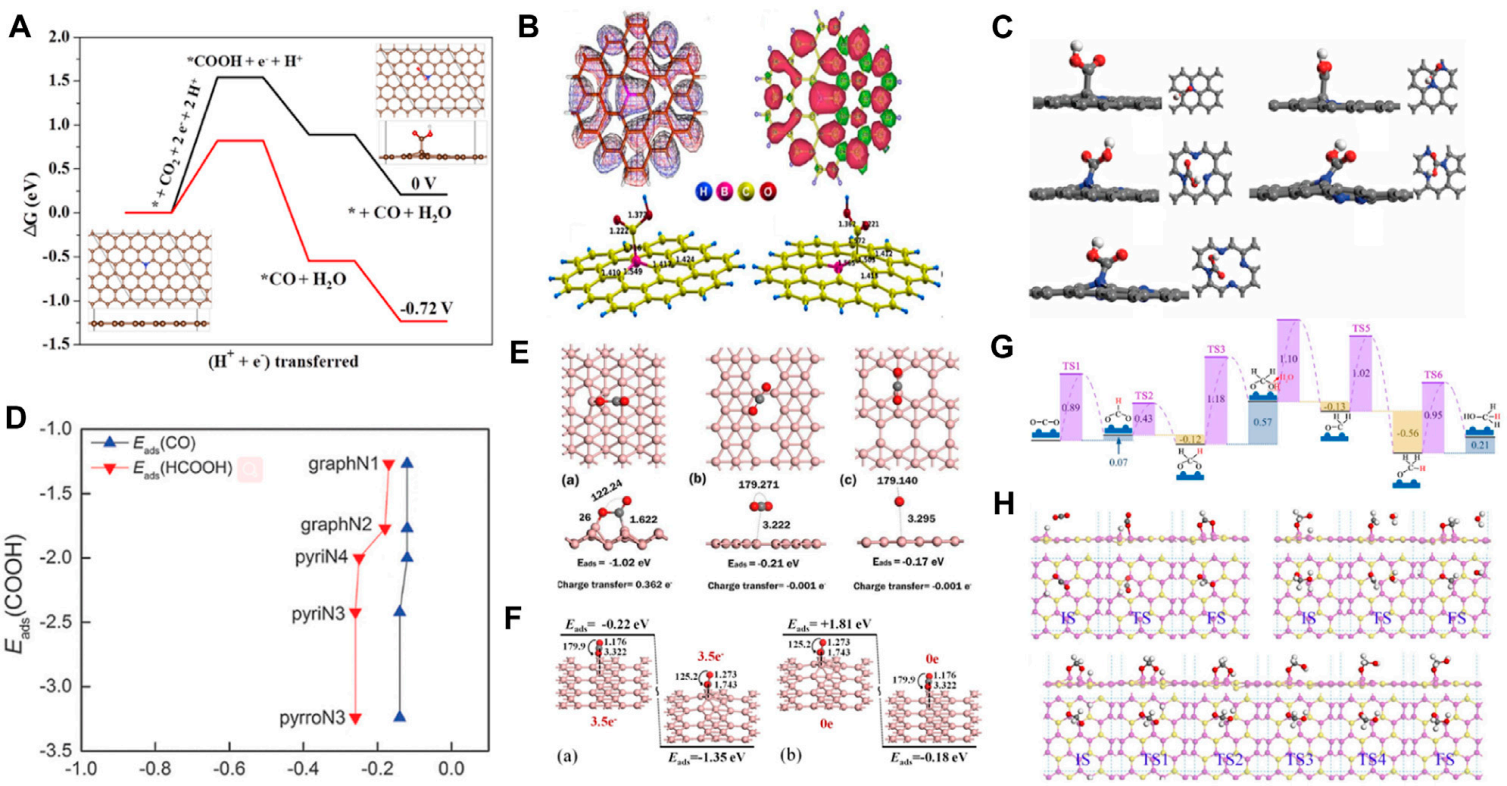
**(A)** The calculated free energy diagram for CO_2_RR to CO on NG-1000 catalyst at 0 V and −0.72 V vs. RHE. (Figure used with permission from Gu et al., 2021). **(B)** Electron configuration of C_41_BH_16_ and adsorption configuration of *COOH on different sites. (Figure used with permission from Phani et al., 2013). **(C)** Structure of COOH species on N-doped graphene. **(D)** The correlation of the adsorption energies of the intermediate species on various N-doped graphenes. (Figure used with permission from Cai et al., 2016). **(E)** The adsorption energy and charge transfer (e−) in the most stable configuration of CO_2_ adsorption on borophene, β_12_ and χ_3_ boron sheets (figure used with permission from Smith et al., 2019). **(F)** Energy change of CO_2_ on borophene by injection and removal of 3.5 extra electrons (figure used with permission from Smith et al., 2017). **(G)** Free energy diagram for “Formate” path and the models of intermediates and the transition states. **(H)** The initial, transition, and final states of the OCHO*, OCH_2_* and OCH_2_OH* on B_2_S (figure used with permission from Sun et al., 2020).

Metal catalysts have developed rapidly in electrocatalysis since Hori et al. used metals for electrocatalytic reactions. However, there are some limitations, such as poor tolerance of metal catalysts in acidic and alkaline environments. In recent years, metal-free materials have emerged as a new class of electrocatalytic materials. Gu et al. demonstrated the potential of producing CO using an N-rich (11.0 wt%) graphene-like carbon electrocatalyst (NG-1000). The C atom next to the graphite-N atom in NG-1000 was the main active species of CO_2_RR. The absorbed COOH* species was considered the potential-limiting step with an overpotential of 0.82 eV (Figure 6A) (Li et al., 2021b). Phani et al. found that the adsorption energy for CO_2_ molecules was higher on boron (B)-doped graphene. Additionally, it was observed that the catalyst promoted the reduction of CO_2_ to formic acid. DFT theoretical calculation showed that B doping led to an asymmetric spin density and activated inert carbon atoms, resulting in higher electrocatalytic activity for the catalyst than graphene (Figure 6B) (Sreekanth et al., 2015). Einaga et al. reported that B-doped diamond (BDD) could catalyze CO_2_ reduction to HCOOH and HCHO. BDD had high stability, and its activity originated from the low binding energy of the *COOH intermediate (Natsui et al., 2018). Scheier et al. studied charge-dependent CO_2_ adsorption in C_60_ and found that C_60+_ achieved the highest CO_2_ adsorption capacity by exposing C_60_ and CO_2_ to the ionizing free electrons by molecular dynamics simulations (Ralser et al., 2016). Cai et al. systematically studied the effect of pyridine N-doped graphene on the electrocatalytic CO_2_RR performance (Liu et al., 2016). The activity of CO_2_ improved as N-doping changed the electronic properties of graphene. The adsorption energies of the COOH species on N-doped graphene were higher than on pristine graphene. However, the weak adsorption energy of CO or HCOOH made desorption easy to produce CO and HCOOH. PyrroN3 possesses the best catalytic performance for CO_2_RR to produce HCOOH due to its lower overpotential of 0.24 V (Figures 6C, D). Borophene is used for electrocatalytic CO_2_RR to produce CH_4_ with a limiting potential of −0.27 V (Qin et al., 2020). The electron-deficient borophene can transfer electrons to CO_2_ and activate it by breaking the π bond. Borophene showed strong activity for CO_2_ with an adsorption energy of −1.02 eV (Figure 6E). Smith et al. calculated the adsorption energy of CO_2_ on borophene nanosheets by adjusting charge density (Tan et al., 2017). The results indicated that the CO_2_ molecule was chemisorbed on the B_3_ site of the neutral and 3.5 e^–^negatively charged borophene. CO_2_ molecules had weak physical adsorption on neutral borophene with adsorption energy in the range of −0.15 eV to −0.19 eV. However, the adsorption energy of CO_2_ molecules on 3.5e^–^negatively charged borophene was in the range of −0.4 eV to −0.8 eV. It was shown that negatively charged borophene could promote CO_2_ molecule capture (Figure 6F). Sun et al. adopted the metal-free B_2_S catalyst to convert CO_2_ into CH_3_OH by DFT calculations (Tang et al., 2020). The thermodynamic and kinetic energy barriers were −0.12 eV and 0.43 eV, respectively. The free energy barrier of the catalyst was about 1/3 of Cu in the metal Cu (211) catalyst, demonstrating high catalytic activity (Figures 6G, H).

In summary, metal SACs and metal-free SACs exhibit high activity, stability, and selectivity in electrocatalytic CO_2_RR compared to typical metal catalysts, significantly reducing the catalytic cost. However, SACs frequently aggregate and form huge clusters due to the high surface free energy. Therefore, SACs have high requirements for their loaded carriers. Therefore, DACs have been introduced in recent years. DACs maintain high atomic utilization and stability (Guan et al., 2020b; Li and Tang, 2021). Moreover, the synergy between two metal atoms significantly improves the catalytic activity. DACs have significant development prospects in electrocatalytic reduction.

## 4 DACs

DACs are two metal atoms (homonuclear or heteronuclear) supported on a carrier to create dimers that catalyze the CO_2_RR and form hydrocarbons or oxygen-containing compounds through interatomic synergy (Das and Arunan, 2019). They increase the active sites and enhance the catalytic activity and selectivity of the process, significantly reducing the theoretical limits of SACs (Zhou et al., 2020b; Liu et al., 2022). The synergistic DACs require selective activation of the reactants on the carrier, the highly active substances degrade or produce by-products if the activation rate is not constant, reducing the efficiency of CO_2_RR (Ying et al., 2021; Ling et al., 2022). DACs are primarily classified as homonuclear DACs or heteronuclear DACs. Due to the compatibility of redox reactions between two metals and changes in the kinetics of the catalytic cycle, heteronuclear DACs exhibit superior catalytic performance than homonuclear DACs in electrocatalysis, resulting in the emerging research on heteronuclear double atoms (Zheng et al., 2020). The structures of DACs are difficult to be accurately regulated in experiments. Therefore, DFT is often used to simulate the active sites of atoms, deduce the route of the catalytic reaction, and calculate the free energy barrier of the reaction. The catalytic performance of CO_2_RR was analyzed to judge the catalyst quality. Therefore, DFT plays a pivotal role in double-atom electrocatalysis.

### 4.1 Homonuclear DACs

**FIGURE 7:**
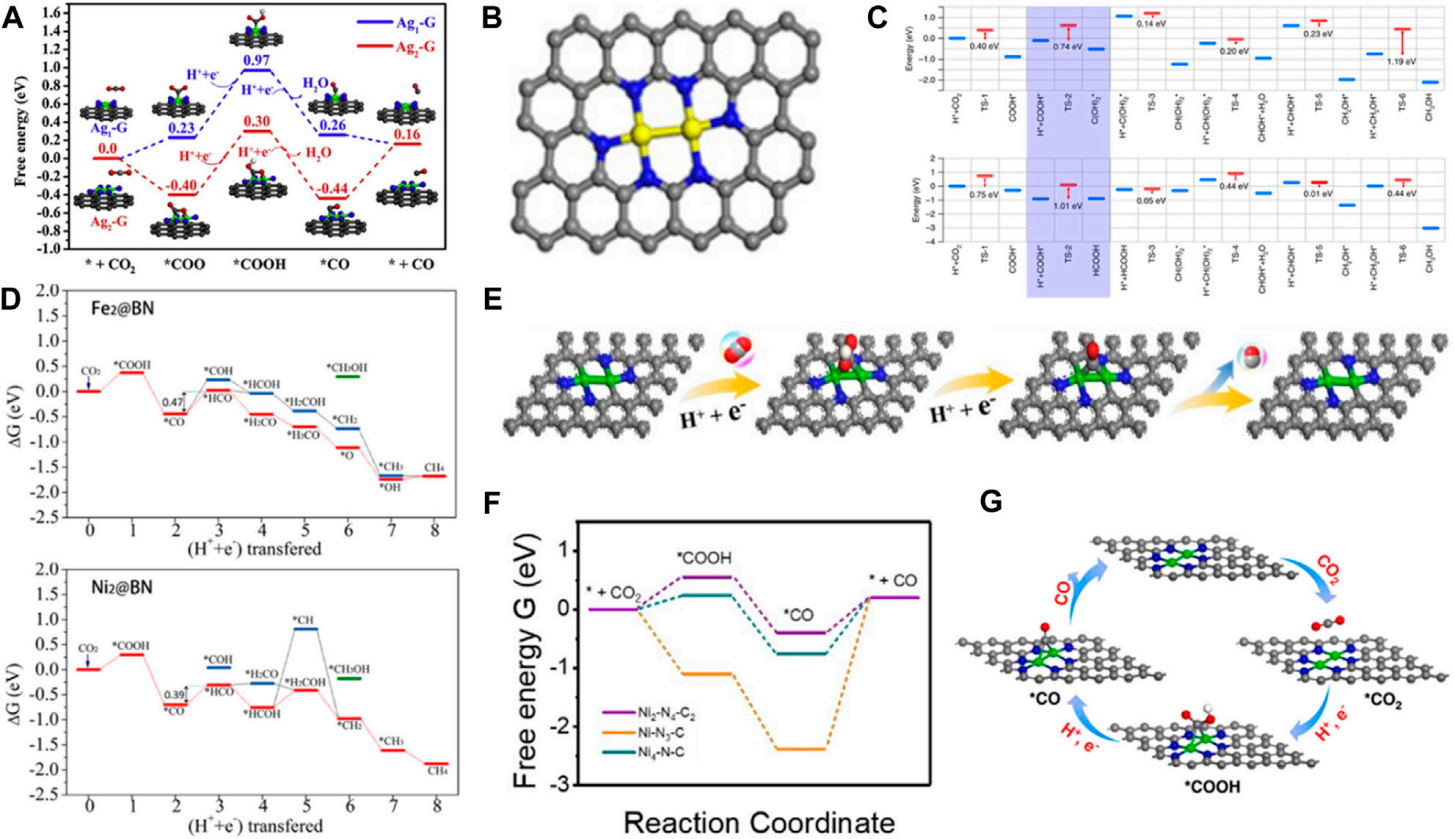
**(A)** Free energy diagrams of CO_2_ reduction on Ag_1_-G and Ag_2_-G catalyst (figure used with permission from Li et al., 2020a). **(B)**The di-atomic models used in EXAFS spectrum fitting (figure used with permission from Cao et al., 2020). **(C)** The CO_2_ reaction paths for Pt_1_/MoS_2_ and Pt_2iii_/MoS_2_ (figure used with permission from Li et al., 2018) **(D)** Free energy profiles of CO_2_ hydrogenation on Fe_2_@BN and Ni_2_@BN nanosheet (figure used with permission from Zhou et al., 2020a) **(E)** Reaction paths for CO_2_ reaction to CO on Ni_2_-N_4_-C_2_ catalyst. Gray, C; red, O; blue, N; while, H; green, Ni. **(F)** Free-energy diagram of CO_2_RR to CO (figure used with permission from Zhang et al., 2022c). **(G)** Scheme diagram of CO_2_ electroreduction to CO on Ni_2_–N_3_ (Ni_2_-NCNT) (figure used with permission from Lu et al., 2023).

Homonuclear metal atoms have similar energy and the same atomic orbitals. In DACs, two identical metal atoms are bonded by a chemical bond, solving the agglomeration phenomenon of SACs due to large surface free energy. DACs increase the content of metal atoms and improve the stability of the catalyst. Zhou et al. (2020c) successfully prepared a double-atom Ag_2_/graphene catalyst (Ag_2_-G) for CO_2_RR (Li et al., 2020b). The two AgN_3_-AgN_3_ sites were firmly anchored to the graphene matrix by the Ag-C bond. The CO_2_ was adsorbed on the two active sites of Ag_2_-G. The formation barrier of *COOH was also reduced, promoting the conversion of CO_2_ into CO with a limit potential of 0.7 eV (Figure 7A). Compared with the Ag_1_-G monoatomic catalyst, DAC showed higher selectivity and excellent catalytic performance. Moreover, the competitive HER was almost completely suppressed. Cao et al. achieved controlled growth of double Ni metal by the metal-N combination strategy in the pyrolysis process (Sun et al., 2020). The DACs reduced the possibility of metal cluster polymerization and showed good activity for electrocatalytic CO_2_ reduction at low Ni content. The electrocatalytic reduction of CO_2_ to CO was effectively achieved by the catalyst (Figure 7B). Zeng et al. systematically studied the synergistic effect of adjacent Pt metal atoms on the catalytic performance of CO_2_ hydrogenation (Li et al., 2018). They prepared Pt/MoS_2_ by replacing Mo of MoS_2_ nanocrystals with Pt atoms. The oxidation rate of Pt gradually decreased with the increased amount of Pt, enhancing the activity of the Pt/MoS_2_ catalyst. DFT calculations indicated that COOH* was the main intermediate obtained. (Figure 7C). Therefore, the Pt_2_/MoS_2_ catalyst can promote CO_2_ conversion to formic acid and methanol. Zhou et al. systematically investigated a series of DACs with various double transition metal atoms supported on defective BN monolayers (TM_2_@BN) (Huang et al., 2020). The onset potentials for CO_2_RR to CH_4_ on various TM_2_@BN nanosheets were built. It was found that Fe_2_@BN and Ni_2_@BN possessed lesser negative onset potentials, and their catalytic performance was better or similar to the Cu (100) and Cu (211) catalysts. In addition, the Fe_2_@BN and Ni_2_@BN exhibited high CO_2_RR activity to produce CH_4_ with limiting potentials of −0.47 and −0.39 V, respectively (Figure 7D). The Ni_2_-N_4_-C_2_ catalyst was synthesized for electrocatalytic CO_2_ reduction to CO with superior activity due to the unique metal-metal bridging structure and proper N coordination number (Cao et al., 2022b). The DFT calculations verified that the d-band center of the catalyst was close to the Fermi level, and more electrons were transferred from the catalyst to the CO_2_ molecules, which was more conducive to CO_2_ adsorption and desorption of *CO intermediates (Figures 7E, F). Double-atoms were anchored on graphitic carbon nitride (g-CN) to investigate their structure stability, CO_2_RR mechanism, and performance (Bui et al., 2021). It was found that Fe_2_@g-CN was a superior electrocatalyst for CO_2_RR with *U*_L_ of 0.58 and 0.54 V for C_1_ and C_2_ products, respectively. The introduction of Fe_13_ clusters into Fe_2_@g-CN caused charge redistribution, which enhanced CO_2_ adsorption and reduced the limiting potentials of CH_4_ formation. Consequently, Fe_2_@g-CN showed higher CO_2_RR activity and selectivity. Lu et al. reported uniformly anchored Ni_2_ dual-atoms on N-doped carbon nanotubes (Ni_2_-NCNT) by modulating the ligands of the precursors for CO_2_RR (Liang et al., 2023). The Ni_2_-N_4_ and Ni_2_-N_3_ DACs models were developed to reveal the activities of the catalysts. A bridged *COOH intermediate at the Ni_2_ catalytic site was formed on the Ni_2_–N_3_ structure, the synergistic effect between two Ni atoms could stabilize the *COOH intermediate, leading to a lower reaction free energy, and Ni_2_–N_3_ exhibited superior CO_2_ reduction activity (Figure 7G). A nitrogen-doped porous carbon anchored homonuclear Fe_2_N_6_ diatomic electrocatalyst has been reported to effectively reduce CO_2_ to CO (Zhao et al., 2022a). The activation of CO_2_ was promoted by the Fe-Fe dual sites. The synergistic effect of two Fe centers further reduces the energy barrier of CO desorption reaction, and the largest energy barrier of CO production was 0.90 eV, which was significantly lower than that at FeN_4_ site (1.03 eV). Therefore, Fe_2_N_6_ was more conducive to the formation of CO products.

### 4.2 Heteronuclear DACs

**FIGURE 8:**
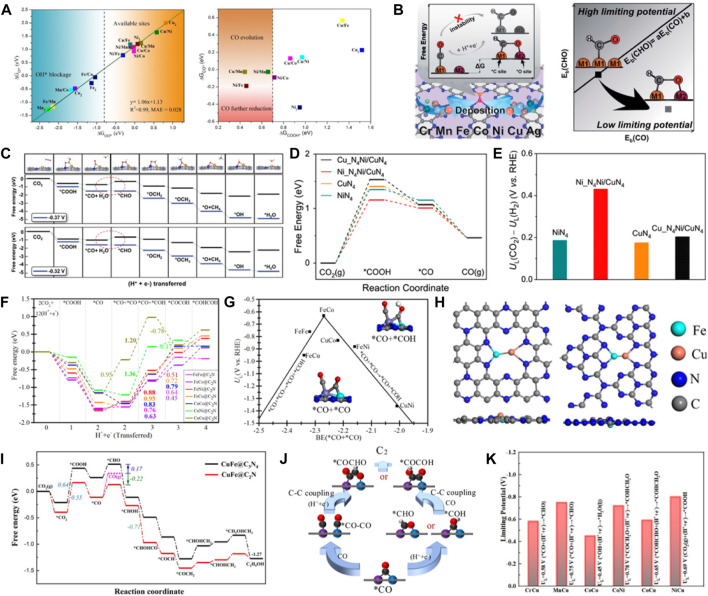
**(A)** Linear diagram of adsorption energy between ΔG_OH*_ and ΔG_O*_ on various DMSCs. COOH* (ΔGCOOH*) and CO* (ΔGCO*) on 9 DMSCs. (Figure used with permission from Li et al., 2020). **(B)** Design of DACs to reduce free energy variation by stabilizing *CHO. **(C)** Simplified schematic of decoupling the scaling relations between binding energies of *CHO and *CO. (figure used with permission from Wang et al., 2020). **(D)** Free energy profiles of the CO_2_RR for four catalysts. **(E)** Difference in limiting potentials for the CO_2_RR and HER (figure used with permission from Lu et al., 2023). **(F)** Gibbs free energy profiles for CO_2_RR toward C2 product. **(G)** Volcano curve between limiting potential (*U*_L_) and binding energy (BE) of co-adsorbed *CO+*CO (figure used with permission from Liu et al., 2021). **(H)** Optimized structures of CuFe@C_2_N and CuFe@C_3_N_4_. **(I)** Free energy profile of CO_2_ reduction to various C2 products. (Figure used with permission from Chen et al., 2021). **(J)** The general two pathway from *CO to C2. **(K)** Limiting potential for the 6 DACs (figure used with permission from Singh et al., 2020).

Heteronuclear DACs have unique structures and electronic properties due to the asymmetric active sites. The symmetry-breaking active centers can cause charge redistribution and more effectively overlap atomic orbitals when activating small fractions. It can strengthen the binding of the reactant molecules at the active sites. Therefore, the catalysts are more conducive to CO_2_ adsorption and activation (He et al., 2022). Luo et al. systematically studied 15 kinds of DACs (ten heteronuclear DACs and five homonuclear DACs) supported on graphene using DFT and dynamic simulation (Luo et al., 2020). Additionally, they screened out three heteronuclear catalysts with high CO_2_RR activity compared to the Au (211), Ag (211), and Cu (211) catalysts. The free energy calculations of the *OH species showed that the active sites of 9 kinds of catalysts were not occupied by the *OH species when the free energy was lower than −0.8 eV (Figure 8A). The adsorption of *COOH and *CO showed that the free energy values of the *COOH intermediate on Cu/Mn, Ni/Mn, and Ni/Fe catalysts were lower than 0.7 eV, exhibiting strong *COOH adsorption. Further hydrogenation and desorption to obtain CO on the catalyst surface were easy. Wang et al. designed 21 kinds of heteronuclear double atoms supported on monolayer C_2_N as effective DACs for CO_2_RR (Ouyang et al., 2020). The analysis of the electronic structures and adsorption configurations of CuMn/C_2_N, CuCr/C_2_N, FeCr/C_2_N, and MnCr/C_2_N showed that they were the most probable catalysts to produce CH_4_ with low overpotential and high selectivity. The C-affinity and O-affinity active sites were formed on the heteronuclear DACs, breaking the transition-metal scaling relations (Figures 8B, C). CuCr/C_2_N and CuMn/C_2_N exhibited the best performance for CH_4_ formation with *U*_L_ of −0.37 V and −0.32 V, respectively. Furthermore, CO_2_ reduction to CO was proposed on the Ni-Cu dual active sites supported on MOF-templated porous carbon (Cheng et al., 2021). The DFT calculations showed that the electronic redistribution and band gap narrowing caused by coordinating Cu with the Ni site enhanced the electron conductivity and decreased the energy barrier for CO_2_ activation. The *COOH formation was the rate-limiting step due to the strengthened interactions between *COOH intermediates and the Ni centers (Figures 8D, E). DACs have been reported in the literature for synthesizing C_2_ products *via* C-C coupling. Fe-Co@C_2_N was predicted as a promising DAC for producing C_2_H_4_ (Liu et al., 2021). The synergistic effect of dual-metal-atom active sites and C_2_N substrate can promote CO_2_ activation (Figure 8F). The study on the CO_2_RR performance of seven DACs found that the FeCo@C_2_N exhibited the lowest limiting potential of −0.63 V for CO_2_RR to produce C_2_H_4_ by *CO+*CO co-binding (Figure 8G). It was revealed that C-affinity was beneficial to the coupling of the C–C bond and C_2_H_4_ formation. Chen et al. reported Cu-Fe atomic pairs anchored on nitrogenized carbon monolayers for electrocatalysis of CO_2_ reduction to C_2_ products with high activity and selectivity (Xie et al., 2021). The coordinated CuFe dimer on the nitrogenized graphene substance prevented metal agglomeration (Figure 8H). It showed strong stability and significantly improved activity for CO_2_ reduction to C_2_ products. Especially, the synergistic effects of metals on CuFe@C_2_N could significantly increase the adsorption ability of the ∗CO species intermediates, exhibiting high activity and selectivity for the CO_2_ reduction to produce C_2_H_5_OH (Figure 8I). Goddard et al. reported a similar study where it was found that the heteronuclear FeCu−grafiN_6_ promoted efficient electron transport and generated C_2_ products at a low limiting potential of −0.68 V (Chen et al., 2020a). Chen et al. conducted a systematic computational screening of double transition metal embedded N-doped graphene for electrocatalysis of CO_2_ reduction to C_2_H_4_ and C_2_H_5_OH (Figure 8J) (Chen et al., 2020b). It was demonstrated that 6 DACs had the stability and electrocatalytic activity for CO_2_RR to produce C_2_ by screening the reaction paths and the limiting potentials for 21 DACs (Figure 8K). Moreover, it was verified that the energy barrier of C-C coupling of the CuCr DAC was much lower than the threshold value (+0.75 eV), exhibiting the lowest limiting potential of −0.58 V for CO_2_RR to obtain C_2_ products. Chen et al. used the heteronuclear diatomic Mo-Se catalyst to explore the reaction of CO_2_ conversion to CO. The formation of COOH* was thermodynamic favorable on MoSA. However, the formation of CO* has a high free energy barrier, and the strongly adsorbed CO* may poisoning the catalyst. After the introduction of SeSA, the free energy of CO* decreased from 1.28 (MoSA) to 1.15 eV (MoSA-SeSA), indicating that the synergistic effect of MoSA-SeSA can significantly promote the generation of CO (Sun et al., 2022). The potential of B, P doped C_2_N monolayer as metal-free catalyst for reducing CO to C_2_H_4_ was explored (Wang et al., 2022). It was shown that B&P/C_2_N catalysts contribute to C-C coupling and prevent catalyst poisoning due to suitable adsorption energy for CO and lower ethylene desorption energy. In addition, B&P/C_2_N catalysts showed high stability. *COCO intermediates were captured by B-N center and transferred to B-P center for C-C coupling and hydrogenation to produce C_2_H_4_. Hydrogen evolution reaction further demonstrated the high selectivity of catalyst for C_2_H_4_ synthesis.

Although DACs have been reported in electrocatalysis in an infinite number of ways, there are significant difficulties in experimental research, the studies on the catalytic mechanism are more one-sided, and the role of the synergy between double atoms at the atomic scale is unknown. Therefore, future studies must focus on efficiently handling experimental obstacles and employing theoretical calculations to investigate synergy mechanisms.

## 5 Electrode potential and solvent effect on electrocatalytic CO_2_RR

**FIGURE 9:**
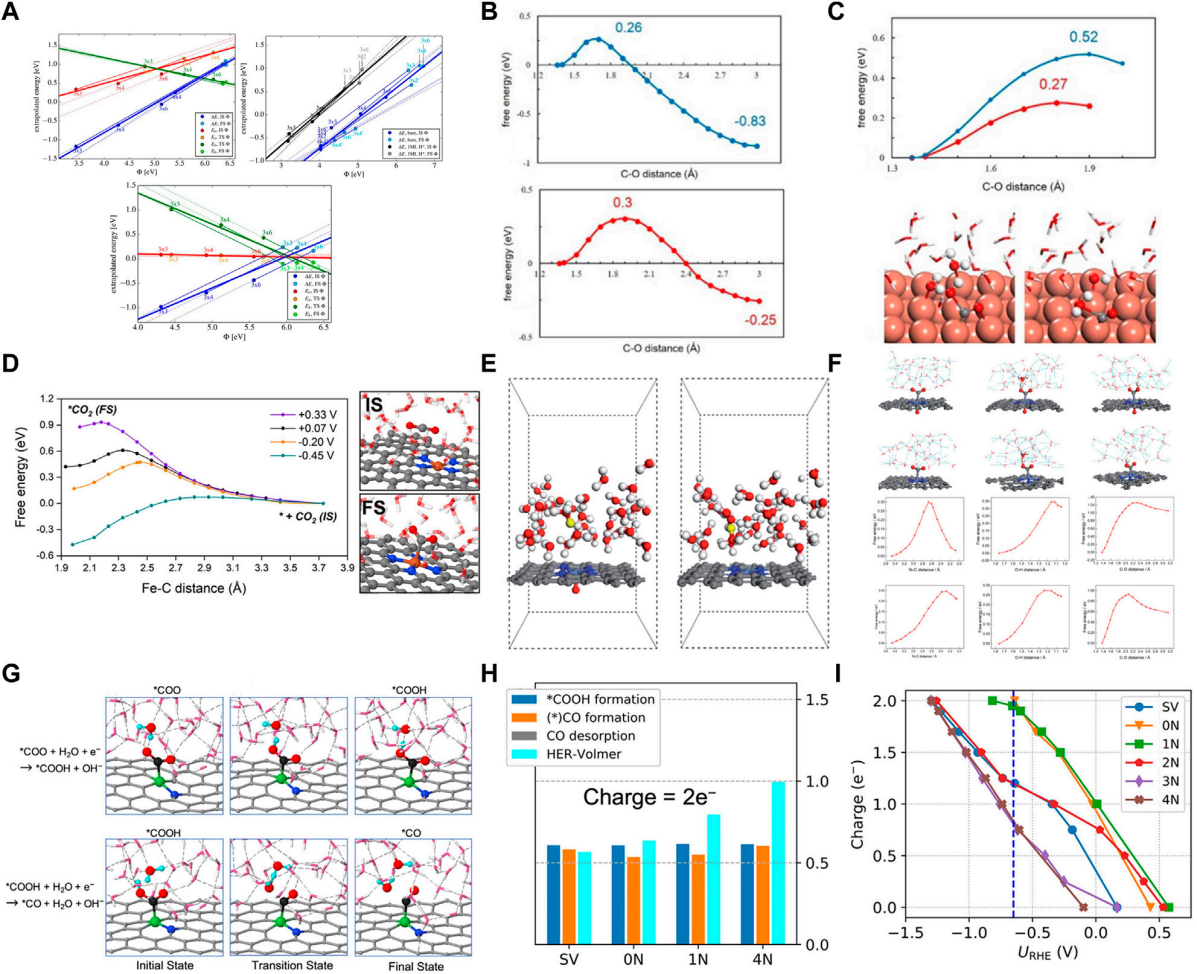
**(A)** Each constant charge barrier/reaction energy is represented by thin solid lines; thin dotted lines stand for beyond these energies. (Figure used with permission from Nørskov et al., 2016) **(B)** Free energy profiles for the C-O_1_ (in blue) and C-O_2_ bond breaking (in red). **(C)** Free energy profiles for the C-O_1_ and C-O_2_ bond breaking and key structures in the C-O_1_ bond breaking. (Figure used with permission from Sun et al., 2012) **(D)** Free energy profiles of CO_2_ adsorption under different potential energy potentials at Fe-N_4_-C/water interface. Reproduced with permission. (figure used with permission from Wang et al., 2022) **(E)** Geometric configuration of NiN_4_–O/C and NiN_4_/C in an aqueous solution. Grey, C; blue, N; light blue, Ni; red, O; white, H; yellow, hydronium. **(F)** Snapshots and reaction energies of initial, transition and final states for the *COOH formation on NiN_4_–O/C and NiN_4_/C by AIMD simulations. (Figure used with permission from Zhou et al., 2021) **(G)** Reaction evolution snapshots of *COOH and *CO formation on 1 N site. Color codes: Ni, Green; C, black; O, red; H, cyan spheres. Dashed lines represent hydrogen bonds. **(H)** Charge = 2e^−^ and **(I)** Charge capacity for *COOH at different sites under the potentials. (Figure used with permission from Liu et al., 2020).

The traditional computational hydrogen electrode (CHE) model assumes that the catalyst is charge-neutral for calculating the energy barrier of a reaction (Andrew et al., 2010). However, the electrochemical reaction usually occurs at the gas-liquid-solid phase interface with a constant electrode potential. The surface of the catalyst is charged, and the electron exchange between the catalyst and the electrode reaches an equilibrium state (Heenen et al., 2020; Zhao and Liu, 2020; Zhao et al., 2022b). The surface charge can significantly change the Fermi level and the electronic structure of the catalyst. Additionally, more electrons accumulate due to the increased charge capacity, significantly favoring the electrochemical reaction (Cao et al., 2023). Therefore, the influence of surface charge and constant potential on catalytic reactions cannot be ignored in heterogeneous electrochemistry. Nørskov et al. determined the constant potential reaction energetics by calculating a single potential barrier in an electrochemical environment and corresponding surface charges in the initial, transition, and final states (Figure 9A) (Chan and Nørskov, 2015). It was also demonstrated that the potential dependence of the activation energy was related to the partial charge transferred in the transition state and predicted by calculating a single constant charge (Chan and Norskov, 2016). Furthermore, the electrode potential significantly affects the elementary steps of proton-electron transfer for electrochemically reducing CO_2_ to CO on the Cu (100) catalyst (Sheng and Sun, 2017). The potential barrier of the electrochemical step decreased with an increase in the electrode potential (Figures 9B, C). Wang et al. probed the CO_2_ proton-electron transfer processes at different potentials using constrained MD sampling and thermodynamic integration (Cao et al., 2022c). And the electrode potential of the system was adjusted by introducing anions and cations (Na^+^, Cl^−^). Subsequently, it revealed the ET-PT decoupling and H bond-assisted CO_2_ activation mechanism. The adsorption of CO_2_ was significantly coupled with the electron transfer from the substrate (Figure 9D). And the electrode potential was essential to achieve efficient activation of CO_2_ adsorption. In addition to the electrode potential, the solvent effect is shown to be another important factor influences the reaction. The H bonding network between the water solvent molecules interacts strongly with the key species adsorbed on the catalyst surface affecting the intermediate stability. The water molecules promote the transfer of H protons and accelerate the reaction. Zhou et al. (2020b) studied the facilitative effect of axial O on CO_2_ activation on NiN_4_ SACs using constrained *ab initio* molecular dynamics (AIMD) with the explicit solvent model. 36 H_2_O molecules and an additional hydrogen atom were introduced into the system to construct the explicit solvent model (Figure 9E) (Hu et al., 2021). Additionally, the last 1,000 samples from the 3–6 ps AIMD simulations were collected to get the potential of mean force to obtain the free energy profile of CO_2_RR and the structures of explicitly solvated intermediates. The constant potential correction method proposed by Chan and Nørskov was used to determine the constant potential of −0.90 V *vs*. SHE. It was found that the H atoms in the solvent environment bonded with the O atoms in the CO_2_ molecule to form *COOH intermediates. Furthermore, the solvent formed H bonds with the adsorbates, the C-O bond was completely broken, and the dissociated OH group was protonated by water molecules, promoting the protonation of CO_2_ to form CO (Figure 9F). Liu et al. investigated six possible active sites on Ni-N_x_C_y_ catalysts for reducing CO_2_ to CO, including Ni embedded in graphene-single vacancies (SV), and x N atoms coordinated with the Ni atom embedded in a divacancy (x = 0, 1, 2, 3, and 4) (Zhao and Liu, 2020). The solvent effect and the applied electrode potential were simulated using six layers of water molecules and net charge, respectively, resolving the catalytic origin due to both factors. The kinetic barriers were evaluated using AIMD and a “slow-growth” sampling approach. The calculations revealed that the multiple H bonds between the solvent water molecules and the polar intermediates improved the intermediate stability (Figure 9G). Moreover, the water molecules facilitated the transfer of protons from water to the intermediates, improving the selectivity of the reaction. Different net charges were introduced at −0.65 V *vs.* RHE. The 0 N, 1 N, and SV sites had nearly two negative charges (2e^−^). However, the 4 N site had nearly one negative charge (1e^–^) for the adsorption of CO_2_. Moreover, the formation barrier for *COOH was lower on 0 N, 1 N, and SV sites. After *COOH formation, 0 N and 1 N sites still had nearly two negative charges (2e^−^) (Figures 9H, I). However, the SV site had only ∼1.2e^–^, resulting in a large formation barrier for the *CO species on the SV site. Therefore, the barriers of electrochemical reactions can be significantly lowered by excessive charge.

## 6 Summary and outlook

SACs have attracted wide interest and have undergone continuous development recently in electrochemical catalysis due to maximum atom utilization and homogeneous active sites. SACs can convert CO_2_ into more valuable products with high electrocatalytic activity and selectivity. Furthermore, developing non-precious metal SACs is highly attractive in reducing reliance on precious metal catalysts. It not only reduces electrochemical costs, but also changes the monolithic nature of precious metal CO_2_RR products, which have been intensively explored by researchers. Recently, metal-free catalysts have been applied to CO_2_RR, exhibiting high activity, stability, and selectivity. However, due to the high free energy of SACs, they can form agglomerates. Therefore, the synergetic effect of DAC circumvents the conventional linear scaling relationship to break through the limitations of SACs. Additionally, DACs show a very high potential to replace SACs in electrocatalysis. Although computational breakthroughs for both SACs and DACs have been made in recent years, there are still many bottlenecks to overcome despite the computational breakthroughs made for SACs and DACs in recent years.

Under practical catalytic conditions, explicit solvation effects and electrode potentials non-negligible factors in catalytic reactions. Therefore, it is necessary to construct an explicit aqueous solvent model and introduce the electrode potential. The kinetic barriers of the reaction were fitted using AIMD and “slow-growth” approaches. They revealed the interaction between the H bond networks and the reactive species and the structure-dependent charge states in the presence of electrode potential, bridging the gap between experiments and computations. In addition, theoretical calculations can be utilized to examine catalytic mechanisms and determine the reaction routes or pathways when experimental methods are insufficient to address related issues. DFT describes the electronic structure and free energy of adsorption for the catalyst, which can be used to determine catalytic performance and reaction potential. DFT has strong directional relevance for mechanism research and performance.
